# Silicon Nanowire Heterojunction Solar Cells with an Al_2_O_3_ Passivation Film Fabricated by Atomic Layer Deposition

**DOI:** 10.1186/s11671-019-2930-1

**Published:** 2019-03-15

**Authors:** Shinya Kato, Yasuyoshi Kurokawa, Kazuhiro Gotoh, Tetsuo Soga

**Affiliations:** 1 Department of Electrical and Mechanical Engineering, Nagoya Institute of Technology, Syouwa-ku, Nagoya-si, Aichi 466-8555 Japan; 2 Department of Materials Process Engineering, Nagoya University, Furo-cho, Chikusa-ku, Nagoya, 464-8603 Japan

**Keywords:** Silicon nanowire, Passivation, Chemical–mechanical polishing, Atomic layer deposition, Solar cell

## Abstract

Silicon nanowires (SiNWs) show a great potential for energy applications because of the optical confinement effect, which enables the fabrication of highly efficient and thin crystalline silicon (c-Si) solar cells. Since a 10-μm-long SiNW array can absorb sufficient solar light less than 1200 nm, the 10-μm-long SiNW was fabricated on Si wafer to eliminate the influence of the Si wafer. On the other hand, Surface passivation of the SiNWs is a crucial problem that needs to be solved to reduce surface recombination and enable the application of SiNWs to c-Si solar cells. In this study, aluminum oxide (Al_2_O_3_) was fabricated by atomic layer deposition for the passivation of dangling bonds. However, owing to a complete covering of the SiNWs with Al_2_O_3_, the carriers could not move to the external circuit. Therefore, chemical–mechanical polishing was performed to uniformly remove the oxide from the top of the SiNWs. A heterojunction solar cell with an efficiency of 1.6% was successfully fabricated using amorphous silicon (a-Si). The internal quantum efficiencies (IQE) of the SiNW and c-Si solar cells were discussed. In the wavelength region below 340 nm, the IQE of the SiNW solar cell is higher than that of the c-Si device, which results in an increase of the absorption of the SiNW cells, suggesting that SiNWs are promising for crystalline-silicon thinning.

## Introduction

**Fig. 1:**
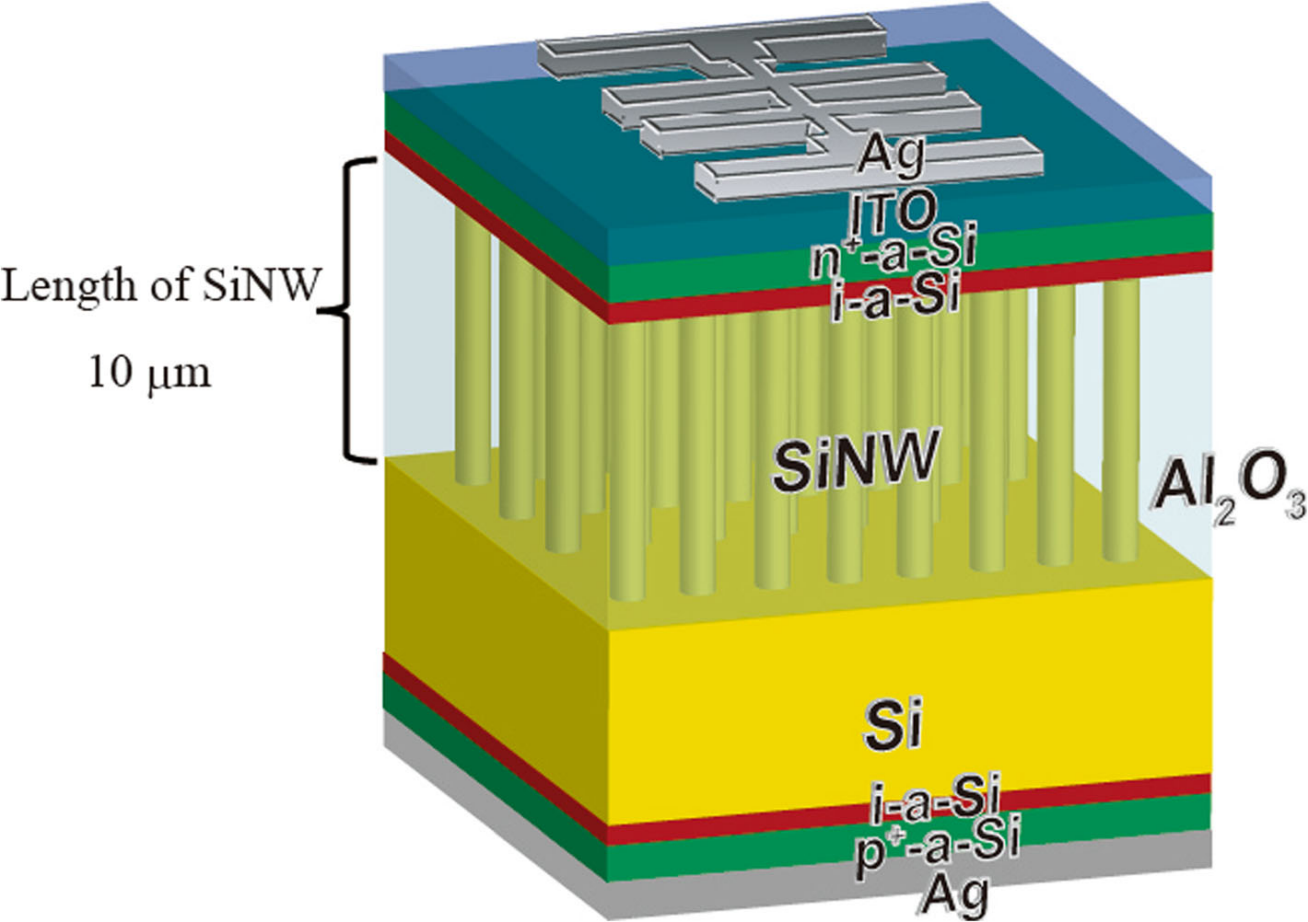
SiNW solar cell structure with Al_2_O_3_

Crystalline silicon (c-Si) solar cells are widely used worldwide because of their high efficiency and abundance [1–9]. To reduce the power generation costs of such solar cells, their efficiency must be increased and their fabrication cost must be reduced. However, the efficiency of c-Si solar cells is close to the theoretical efficiency limit and further improvement is difficult because the open-circuit voltage (*V*_oc_) is limited by Auger recombination [10, 11]. Creating very thin c-Si films is an effective way to improve *V*_oc_, but extremely thin c-Si solar cells exhibit a low short-circuit current density (*I*_sc_) because of their low absorption coefficient [12, 13]. Recently, silicon nanowires (SiNWs) have attracted considerable attention because they exhibit a strong optical confinement effect that is essential for trapping light in solar cells [14–21]. In our previous experiments, we succeeded in evaluating the optical properties of SiNWs by peeling them from silicon wafers using polydimethylsiloxane [22]. A 10-μm-long SiNW array can absorb sufficient light, which indicates that SiNWs can reduce the thickness of c-Si solar cells. Since it is difficult to fabricate self-standing SiNW array, the Si wafer is needed. In this study, we focused on the fabrication of 10-μm-long SiNW arrays on the Si wafer. Therefore, to maximize the absorption in the wavelength below 1200-nm region by 10-μm-long SiNW arrays, the influence of Si wafer can be eliminated. On the other hand, to apply SiNWs to solar-cell structures, it is necessary to fabricate a passivation film on their surface to reduce surface recombination. We found that SiNWs exhibit a high aspect ratio, so it is difficult to fabricate a passivation film by chemical vapor deposition. Therefore, the passivation film was fabricated on the SiNW surface by atomic layer deposition (ALD) [23, 24]. On the other hand, SiNW arrays containing Al_2_O_3_ cannot be peeled from the silicon wafer due to the improved mechanical strength. Moreover, the carriers cannot move to the external circuit because of the insulating Al_2_O_3_ film. In this study, we propose a new structure (shown in Fig. 1) in which 10-μm-long SiNWs are fabricated on a Si wafer.

To form a contact between the SiNWs and a-Si, the Al_2_O_3_ present on the top of the SiNWs was removed by chemical–mechanical polishing (CMP) and etching. The influence of Al_2_O_3_ etching on the properties of the solar cells was investigated.

## Methods

### Fabrication of SiNW Arrays and Al_2_O_3_

A p-type Si (100) wafer (8–10 Ω cm, 550 μm) was immersed in hydrofluoric acid (HF) solution with AgNO_3_ to deposit silver particles. The Si wafer was chemically etched, using 4.8 M HF and 0.15 M H_2_O_2_ at room temperature, and subsequently added into an HNO_3_ solution to remove the silver films. Finally, the oxide layer present on the prepared SiNW array was removed using the HF solution. SiNWs with lengths of 10, 15, and 20 μm were fabricated by changing the etching time. Since the space between the SiNWs is large, silica particles with a diameter of about 80 nm (dispersed in an ethanol solution) were filled into the space between the wires. Then, 66-nm-thick Al_2_O_3_ was deposited by ALD to passivate the dangling bonds. Field-emission scanning electron microscopy (FE-SEM, JEOL JSM-7001F) was applied to examine the structure of the prepared SiNW arrays.

### Removal of Al_2_O_3_ on the Top of SiNWs

**Fig. 2:**
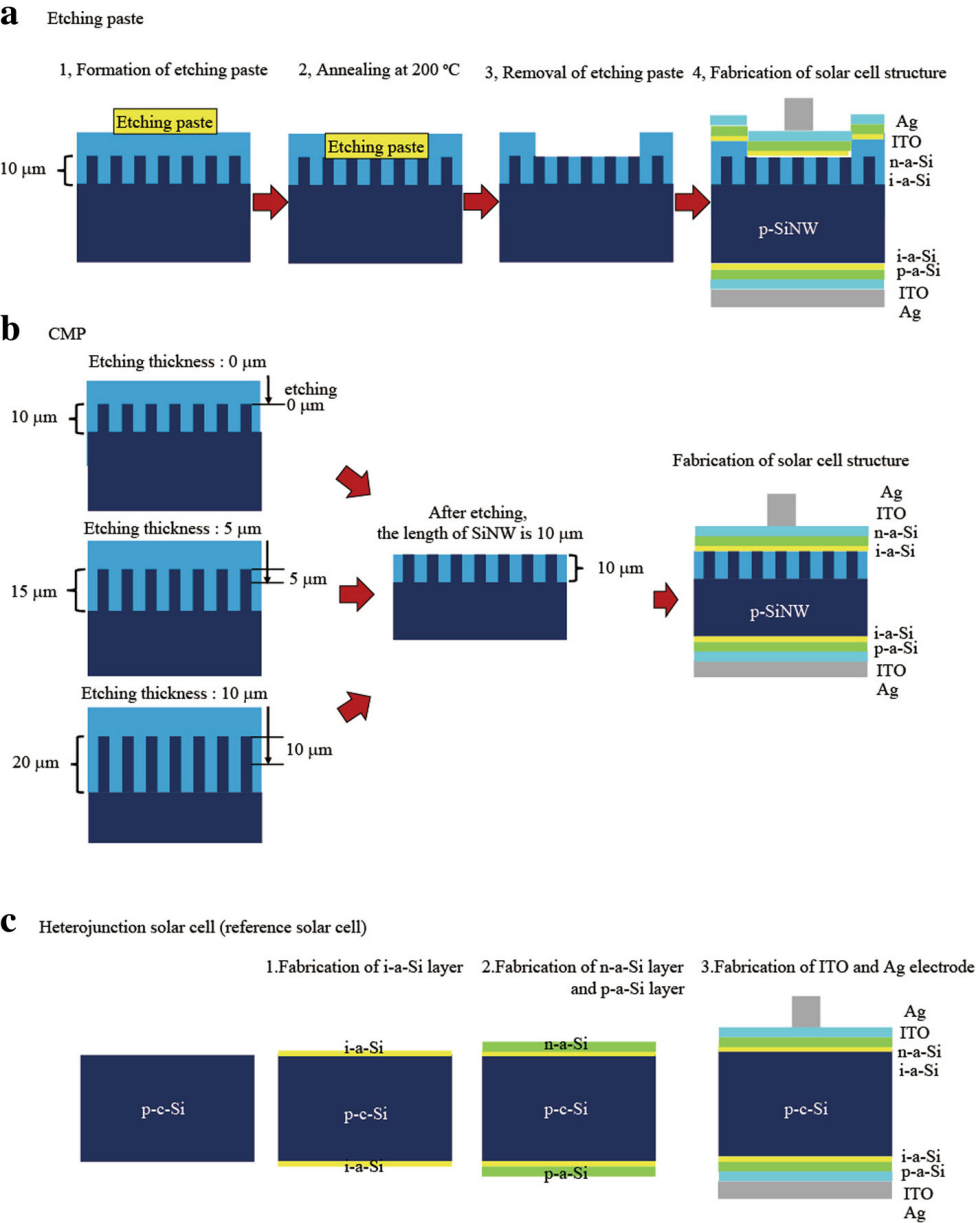
Al_2_O_3_ etching procedure and fabrication procedure of solar cell: **a** etching paste and **b** CMP. **c** Heterojunction solar cell (reference solar cell)

Next, an etching paste and the CMP method were applied to remove the top of the SiNWs and the Al_2_O_3_ on them. Figure 2a shows the Al_2_O_3_ etching procedure using an etching paste. The etching paste was formed on the Al_2_O_3_ layer, followed by annealing to remove it. Finally, the etching paste was removed. In the case of CMP, the detailed process is shown in Fig. 2b. With the fabricated solar cell structure, the length of the SiNW array remained constant at 10 μm, and therefore, the etching thickness was changed by changing the initial length of the SiNW arrays. When the initial length of the SiNWs was 10 μm, the etching was stopped at the top of the nanowires (etching thickness 0 μm, the length of SiNW 10 μm, the thickness of remaining Si wafer 540 μm), which means that the Al_2_O_3_ above the SiNWs was only etched. For an initial SiNW length of 15 μm, the etching length was defined as 5 μm, including the 5-μm SiNWs and Al_2_O_3_ (etching thickness 5 μm, the length of SiNW 10 μm, the thickness of remaining Si wafer 535 μm). When the etching length was defined as 10 μm, the initial length was 20 μm (etching thickness 10 μm, the length of SiNW 10 μm, the thickness of remaining Si wafer 530 μm).

### Fabrication of the Solar Cell Structure

Figure 1 shows the solar-cell structures fabricated herein; the heterojunction structure of a-Si and Si was adopted. Fabrication procedure and condition of the heterojunction structure are the same as the SiNW solar cell and reference solar cell in Fig. 2. In the case of reference solar cells, the p-type Si (100) wafer (8–10 Ω cm, 550 μm) was used without SiNW. A double heterojunction was formed by depositing an i-type hydrogenated amorphous silicon layer (i-a-Si:H, thickness 5 nm), an n-type a-Si:H layer (thickness 10 nm), and a p-type a-Si:H layer (thickness10 nm) via plasma-enhanced chemical vapor deposition (PECVD). Indium tin oxide (ITO) (thickness 80 nm) and an Ag grid were used to fabricate the front electrode. The reflectance of the solar cells was measured in the ultraviolet–visible–near-infrared region. Quasi-steady-state photoconductance (QSSPC, Sin-ton Instruments) experiments were carried out to measure the minority carrier lifetime of the SiNWs. The SiNW solar cells were also characterized by illuminated current–voltage (*I–V*) and quantum efficiency measurements. The parameters of a reference solar cell fabricated on the same wafer without any treatment are shown in Table 1.

**Table 1:** Characteristics of a reference solar cell fabricated on the same wafer without any treatment

	Eff (%)	FF	*V*_oc_ (V)	*I*_sc_ (mA/cm^2^)
Ref solar cell	16.02	0.75	0.66	32.61

## Results and Discussion

**Fig. 3:**
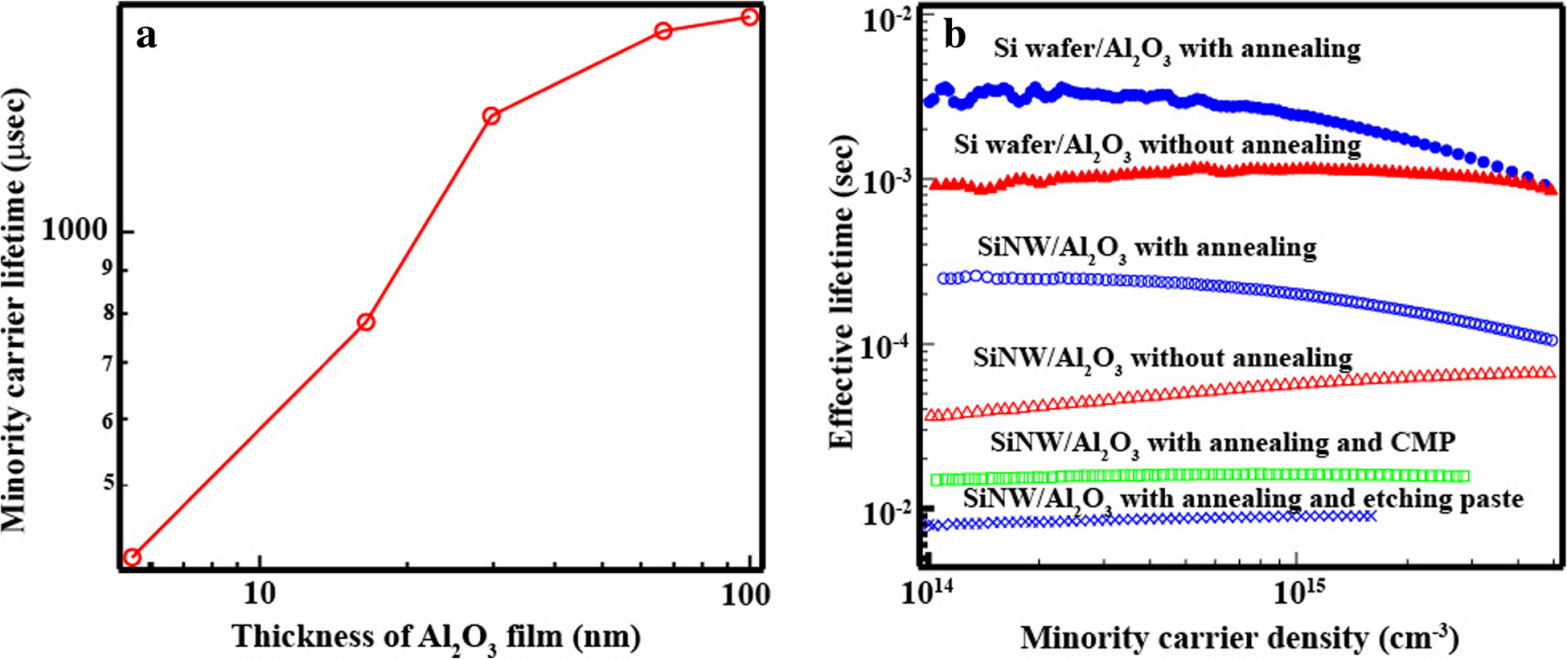
**a** The minority carrier lifetime of Si wafer/Al_2_O_3_ as the function of the Al_2_O_3_ film thickness. **b** The minority carrier lifetime of each sample as the function of the minority carrier density

**Fig. 4:**
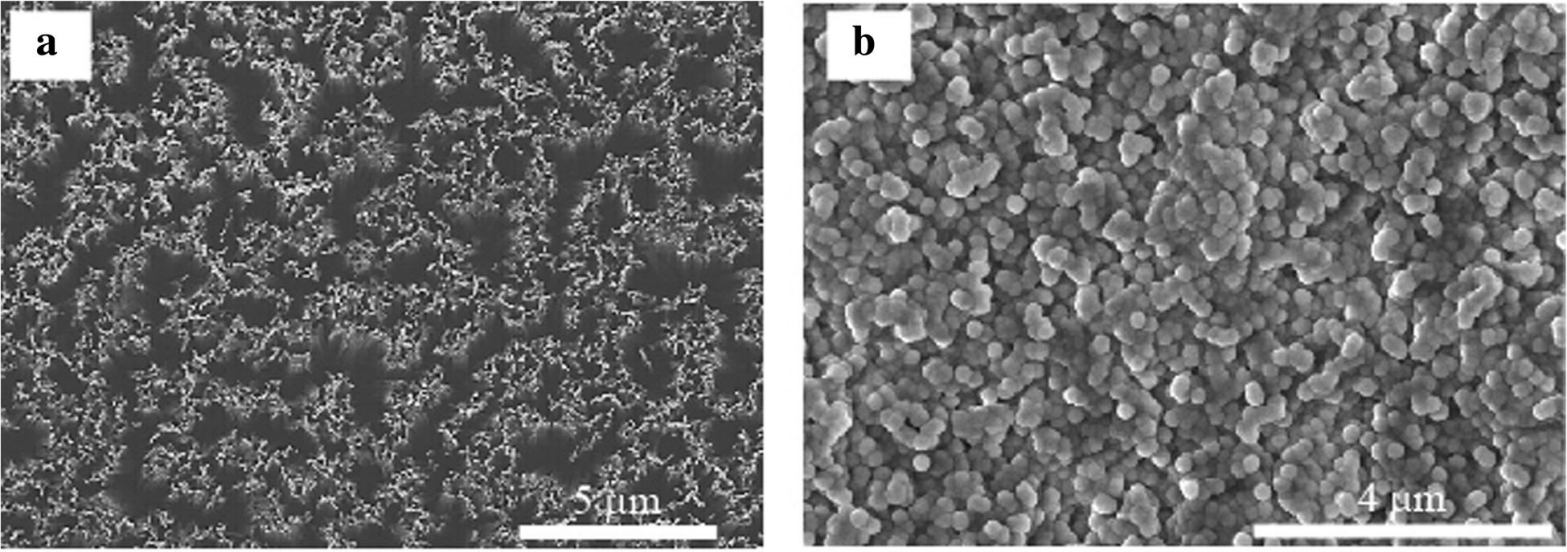
Top views of SEM images of SiNWs **a** without and **b** with Al_2_O_3_

The carrier lifetime for the SiNW array without Al_2_O_3_ could not be measured by QSSPC. Several defects were present on the SiNW surface; these are related to dangling bonds that can lead to a considerable recombination of minority carriers. To passivate the SiNW surface, Al_2_O_3_ was deposited by ALD, as shown in Fig. 3b, with the Al_2_O_3_ deposit being embedded into the SiNW array without space. If there is space in the SiNW/Al_2_O_3_, this film is easily broken by CMP. Moreover, the lifetime Si wafer with Al_2_O_3_ increased with the increasing in the thickness of the Al_2_O_3_ and it tended to be constant from 66 nm as shown in Fig. 4a. From these results, the thickness of the Al_2_O_3_ layer was set to 66 nm. Figure 4b shows the minority carrier lifetime of each sample as a function of minority carrier density. The minority carrier lifetime of SiNW with Al_2_O_3_ increased drastically to 65 μs (Fig. 4). Since the dangling bonds were modified by the Al_2_O_3_, the density of defects decreased. Furthermore improving the minority carrier lifetime of SiNW/Al_2_O_3_, annealing in forming gas (FG) was conducted and the carrier lifetime was improved to 157 μs. When the carrier lifetime of Si wafer/Al_2_O_3_ as a function of carrier density was considered, the trend of that with and without annealing are different. In the region of low carrier density, the carrier lifetime increased by the negative fixed charge. On the other hand, the minority carrier lifetime without annealing decreased due to the becoming dominant of Shockley-Read-Hall recombination. Since the negative fixed charge influences the formation of the band bending at the interface between Al_2_O_3_ and Si surface, the recombination at Si surface can be reduced [25]. We can obtain the information about the existence of negative fixed charge by trend of carrier lifetime as a function of carrier density. Therefore, we found that SiNW/Al_2_O_3_ after annealing was improved by the negative fixed charge. Although SiNWs were completely covered by Al_2_O_3_, the carriers did not move to the external circuit. Thus, to fabricate the solar cell structure, the Al_2_O_3_ present on the top of the SiNWs must be removed using an etching paste and applying the CMP technique.

**Fig. 5:**
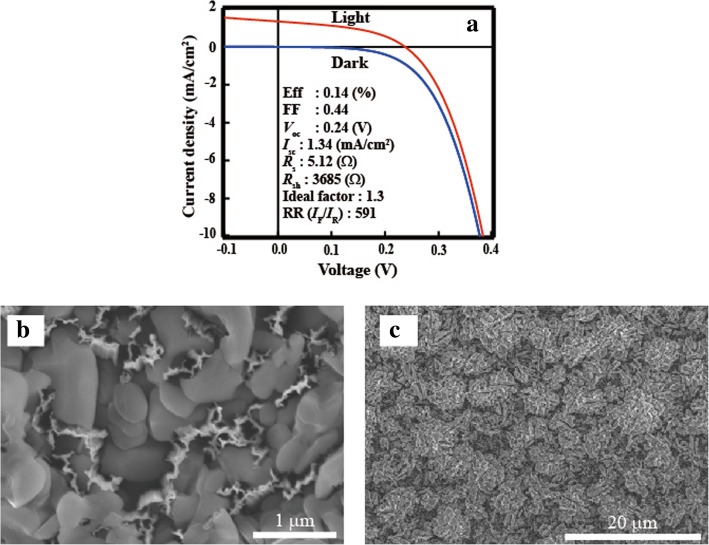
**a** *I–V* characteristics of a SiNW solar cell with Al_2_O_3_ removed using an etching paste. **b** High-magnification top view of the SEM image of SiNWs with Al_2_O_3_ after using an etching paste. **c** Low-magnification top view of the SEM image of SiNWs with Al_2_O_3_ after using an etching paste

Firstly, an etching paste was used to remove the Al_2_O_3_ from the top of the SiNW array. After etching, the heterojunction-solar-cell structure was fabricated by forming an n-a-Si/i-a-Si/p-SiNW/i-a-Si/n-a-Si system. Figure 5a shows the *I–V* characteristics of the SiNW solar cell and the solar cell parameter, series resistance (*R*_s_), shunt resistance (*R*_sh_), ideality factor, and rectification ratios (RR). RR is defined as *I*_F_/*I*_R_, where *I*_F_ (at 0.5 V) and *I*_R_ (at − 0 .5 V) denote the current at forward and reverse bias respectively. The photovoltaic effect was observed for the SiNW solar cell containing Al_2_O_3_, and the result shows the removal of Al_2_O_3_ from the top of the SiNWs. However, the efficiency is low (0.14%) because of the low short-circuit current (*I*_sc_) and open-circuit voltage (*V*_oc_) values. In the case of *V*_oc_, the carrier lifetimes of SiNWs with Al_2_O_3_ decreased to 9 μs after using the etching paste. Figure 5b shows a high-magnification top view of the SEM image of a SiNW array with Al_2_O_3_ after etching. The area in which the SiNWs are exposed is small, and the number of carriers that can be taken out has decreased. Figure 5c shows the low-magnification top view of the SEM image. Since the etching proceeds non-uniformly and the shape before etching was already non-uniform, the non-uniformity of Al_2_O_3_ increases after etching. We found that it is difficult to remove the Al_2_O_3_ uniformly using the etching paste, but to improve the *I*_sc_ of SiNW solar cells, a uniform etching is required.

**Fig. 6:**
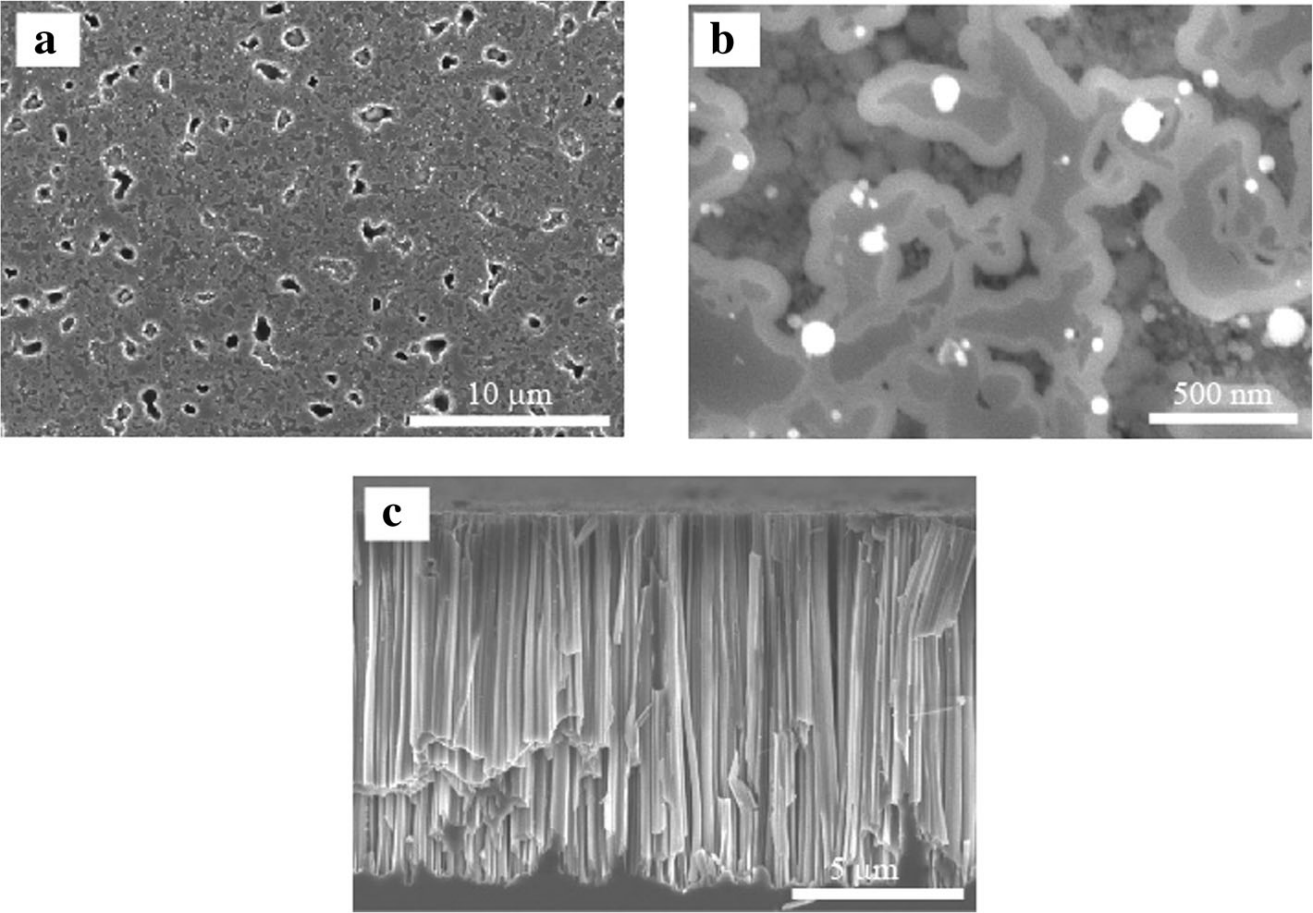
**a** Low-magnification top view of the SEM image of SiNWs with Al_2_O_3_ after CMP. **b** High-magnification top view of the SEM image of SiNWs with Al_2_O_3_ after CMP. **c** Cross-sectional view of the SEM image of SiNWs with Al_2_O_3_ after CMP

CMP was performed to uniformly etch the Al_2_O_3_ deposited on the SiNWs. Figure 6a and b shows the top-view SEM image of SiNWs with Al_2_O_3_ after CMP. First, the SiNW array did not break after CMP, indicating that the mechanical strength of the SiNW array with Al_2_O_3_ is improved by embedding the space between SiNWs. Since CMP can uniformly etch Al_2_O_3_, the top of the SiNW/Al_2_O_3_ film became flat.

**Fig. 7:**
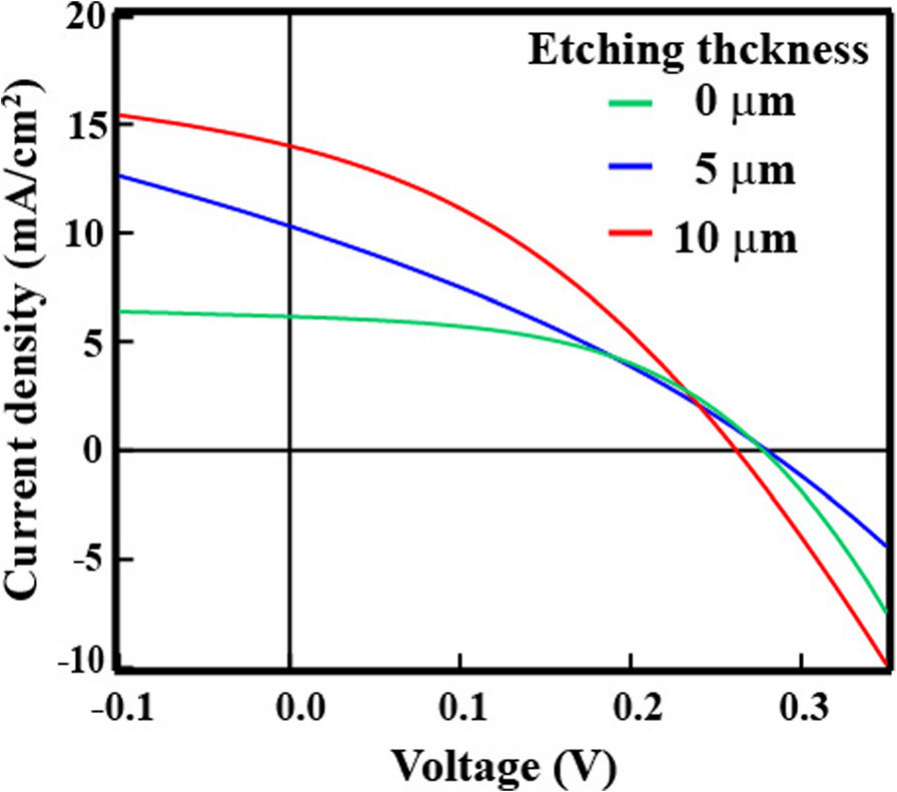
**a** *I–V* characteristics of SiNW solar cells with Al_2_O_3_ removed by CMP

After CMP, the heterojunction-solar-cell structure was fabricated by forming an n-a-Si/i-a-Si/p-SiNW/i-a-Si/n-a-Si using PECVD system. Figure 7 shows the *I–V* characteristics of SiNW solar cells with etching thicknesses of 0, 5, and 10 μm and the solar cell parameter, *R*_s_, *R*_sh_, ideality factor, and RR are listed in Table 2. For an etching thickness of 0 μm (when the top of the SiNWs was observed, etching was halted), the photovoltaic effect was confirmed, with a conversion efficiency of 0.8%. *I*_sc_ of 6.11 mA/cm^2^ was observed. Although the *I*_sc_ value increased compared with the results obtained for the etching paste, it is still a low value. The top of the SiNW arrays was aggregated by surface tension in Fig. 4a. Since a part of the SiNWs did not have contact to the a-Si layer, the carriers moved to the external circuit with difficulty. To improve the contact area, the etching thickness was increased to 5 μm, and the *I*_sc_ increased to 10.3 mA/cm^2^. At an etching thickness of 10 μm, the *I*_sc_ improved to 14.0 mA/cm^2^. As the aggregated SiNW arrays were removed, the contact area between SiNW and a-Si increased. On the other hand, an extremely low *V*_oc_ of 0.3 V was obtained. The minority carriers were measured after CMP, and the minority carrier lifetime decreased drastically from 157 to 19 μs because the passivation quality of the Al_2_O_3_ deposit decreased by CMP. Since the minority carrier lifetime in the region of low minority carrier density declined after CMP, the negative fixed charge decreased. The recombination center on the SiNW surface increased leading to low *V*_oc_. Furthermore, in the case of wires, the carrier mobility is lowered, because of the scattering of carriers on the surface, and the conductivity is lowered. Although these results indicated that negative fixed charge might be reduced by CMP, the mechanism still is unclear. On the other hand, when the *R*_s_, *R*_sh_, ideality factor, and RR of the etching paste and the CMP result was compared, each parameter of the etching paste is better than that of CMP. Since *R*_s_ of CMP is greater than that of etching paste and *R*_sh_ of CMP is lower than that of etching paste, contamination might remain on the top of SiNW leading to preventing good contact between SiNW and a-Si. Therefore, a further study is required to investigate the improvement of the passivation quality for enhancing the *V*_oc_ and *I*_sc_ of solar cells.

**Table 2:** Characteristics of SiNW solar cells with Al_2_O_3_ removed by CMP

Etching thickness	Eff (%)	FF	*V*_oc_ (V)	*I*_sc_ (mA/cm^2^)	*R*_s_ (Ω)	*R*_sh_ (Ω)	Ideality factor	RR *I*_F_/*I*_R_
0 μm	0.82	0.48	0.28	6.11	7.62	1693	1.54	160
5 μm	0.87	0.30	0.28	10.3	8.77	1647	1.72	102
10 μm	1.30	0.36	0.26	14.0	8.39	1654	1.65	120

**Fig. 8:**
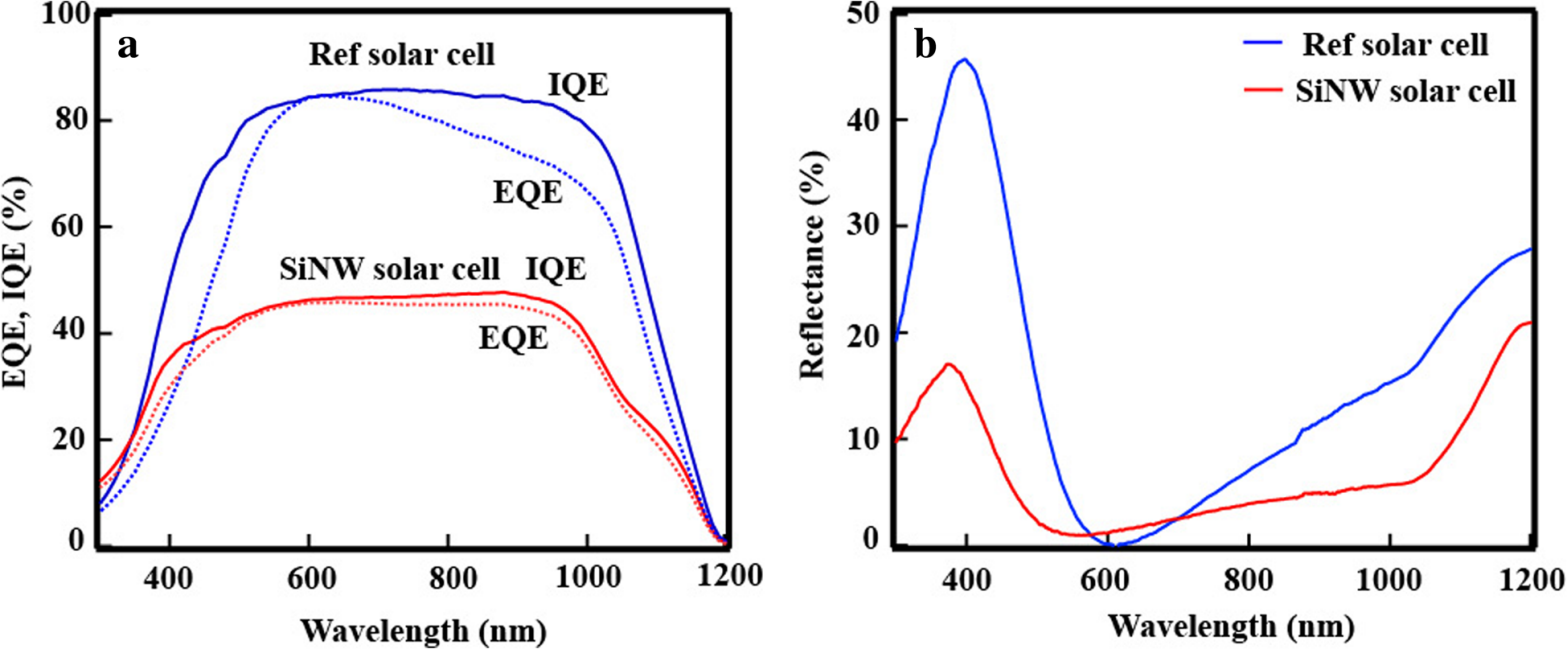
**a** EQE and IQE of a SiNW solar cell and a reference solar cell. **b** Reflectance of a SiNW solar cell and a reference solar cell

The quantum efficiency of 10-μm-long SiNW and c-Si solar cells were compared. In the case of the external quantum efficiency (EQE), the intensity of the SiNW solar cell is mostly lower than that of the c-Si solar cell in Fig. 8a. However, the EQE of SiNW solar cell was improved in the region from 300 to 500 nm. Figure 8b shows the reflectance of the SiNWs and c-Si solar cells, and it can be observed that the reflectance of the SiNWs device is lower than that of the c-Si one, particularly in the short wavelength region (from 300 to 500 nm) where it is drastically decreased. Although the reflectance of the SiNW solar cell is lower than that of the c-Si solar cell, the EQE of the SiNW device in other regions is lower than that of the c-Si solar cell. Since long wavelength region of light was absorbed in the bottom of SiNWs, the EQE of SINW solar cell decreased. The internal quantum efficiencies (IQE) of the SiNW and c-Si solar cells were discussed to eliminate the influence of the reflectance. The wavelength region in which the IQE of the SiNW solar cell is higher than that of the c-Si solar cell decreased. In the wavelength region below 340 nm, the IQE of the SiNW device is higher than that of the c-Si solar cell, which results in an increase of the absorption of the SiNWs. The increase of absorption is caused by a light-trapping effect rather than the optical cavity effect. [26, 27] In order to obtain the optical cavity effect using SiNW, the diameter and position of SiNW should be controlled. Since the diameter and position of SiNW fabricated by MAE was random, it is difficult to obtain the optical cavity effect using the SiNW. On the other hand, the random structure of SiNW can have strong light-trapping effect, suggesting that SiNWs fabricated by MAE are promising for crystalline-silicon thinning.

## Conclusion

Surface passivation of SiNWs is crucial for their application in solar-cell devices. Al_2_O_3_ was fabricated by ALD to passivate the dangling bonds. Since ALD can deposit Al_2_O_3_ over the entire SiNWs, the carrier cannot move to the external circuit. In this study, an etching paste and the CMP technique were applied to etch Al_2_O_3_ from the top of the SiNWs. With the etching paste, SiNW solar cells with 0.14% efficiency were successfully obtained. However, since the SiNW array was aggregated by surface tension, the contact area between SiNWs and a-Si was small, leading to a low *I*_sc_. To further improve the efficiency, the etching thickness was increased, and the efficiency could be improved to 1.6% by increasing *I*_sc_. In the case of the EQE, the intensity of the SiNW solar cell is lower than that of the c-Si solar cell. Since reflectance in short wavelength region from 300 to 500 nm is drastically decreased, the EQE was improved. The IQEs of the SiNW and c-Si solar cells were discussed to eliminate the influence of the reflectance. In the wavelength region below 340 nm, the IQE of the SiNW device is higher than that of the c-Si solar cell, which results in an increase of the absorption of the SiNWs, suggesting that SiNWs are promising for crystalline-silicon thinning.

## Abbreviations

Al_2_O_3_: Aluminum oxide
CMP: Chemical–mechanical polishing
EQE: External quantum efficiency
*I*_F_: The current at forward bias
IQE: Internal quantum efficiency
*I*_R_: The current at reverse bias
*I*_sc_: Short-circuit current
*I-V*: Current–voltage
RR: Rectification ratios
*R*_s_: Series resistance
*R*_sh_: Shunt resistance
SiNW: Silicon nanowire
*V*_oc_: Open-circuit voltage
